# Urinary tract infections in older adults: associated factors for extended-spectrum beta-lactamase production

**DOI:** 10.3389/fmicb.2024.1384392

**Published:** 2024-05-09

**Authors:** Sena Alkan, Ilker Inanc Balkan, Serkan Surme, Osman Faruk Bayramlar, Sibel Yildiz Kaya, Ridvan Karaali, Bilgul Mete, Gokhan Aygun, Fehmi Tabak, Nese Saltoglu

**Affiliations:** ^1^Department of Infectious Diseases and Clinical Microbiology, Istanbul University-Cerrahpasa, Cerrahpasa Medical Faculty, Istanbul, Türkiye; ^2^Department of Medical Microbiology, Institute of Graduate Studies, Istanbul University-Cerrahpasa, Istanbul, Türkiye; ^3^Department of Public Health, Bakirkoy District Health Directorate, Istanbul, Türkiye

**Keywords:** urinary tract infection, extended-spectrum beta-lactamase, *Escherichia coli*, *Klebsiella pneumoniae*, associated factors

## Abstract

**Objective:**

Urinary tract infections (UTIs) due to extended-spectrum beta-lactamase (ESBL) producing *Escherichia coli* and *Klebsiella pneumoniae* are among the leading causes of morbidity and mortality in older adults. Identifying associated factors for ESBL production may contribute to more appropriate empirical treatment.

**Materials and methods:**

This was a prospective observational study. Hospitalized patients of age > 65 with community-onset or hospital-acquired upper UTI due to *E. coli* or *Klebsiella pneumoniae* were included. A multivariate analysis was performed.

**Results:**

A total of 97 patients were included. ESBL prevalence among UTIs with *E. coli* or *Klebsiella pneumoniae* was 69.1% (*n* = 67). CRP values at the time of UTI diagnosis were found to be significantly higher in the ESBL-producing group (*p* = 0.004). The multivariate analysis revealed that male gender (OR: 2.72, CI: 1.02–7.25), prior recurrent UTI (OR: 3.14, CI: 1.21–8.14), and the development of secondary bacteremia (OR: 4.95, CI: 1.03–23.89) were major associated factors for UTI in older adults due to ESBL-producing *E. coli* and *Klebsiella pneumoniae.*

**Conclusion:**

Severe UTI in older men with a history of recurrent UTI may be a warning to the clinician for ESBL production in the setting of high ESBL prevalence. Carbapenems may be prioritized in the empirical treatment of patients with known risk factors for ESBL.

## Introduction

Urinary tract infections (UTIs) are among the most common infectious diseases in the geriatric population and account for 25% of all infections (Curns et al., 2005). The most frequently isolated microorganisms in UTI are *Escherichia coli* (*E. coli*) (75–95%), followed by *Klebsiella pneumoniae*. Among these species, extended-spectrum beta-lactamase (ESBL)-producing strains are increasing worldwide, causing infections not only in the hospital but also in the community setting (Saltoglu et al., 2015; Lob et al., 2016).

ESBL-producing isolates are resistant to most beta-lactams as well as to other classes of antibiotics (Schwaber et al., 2005). As a result, more patients face the risk of receiving inadequate empirical therapy, leading to an increase in morbidity, mortality, and healthcare costs, as well as longer hospital stays and readmissions (Mac Vane et al., 2014; Bischoff et al., 2018; Vallejo-Torres et al., 2018). On the other hand, overuse of antimicrobial agents, particularly carbapenems, such as meropenem and imipenem, perpetuates the antimicrobial resistance problem by promoting the selection of multidrug-resistant pathogens.

Several studies have been conducted to investigate the associated factors for ESBL-producing *E. coli* and *Klebsiella pneumoniae* UTI. However, there are a limited number of studies demonstrating associated factors for ESBL in older adults with UTIs. In this study, we aimed to determine associated factors for ESBL production in UTIs with *E. coli* and *Klebsiella pneumoniae* among older adults. By doing so, we aim to contribute to antimicrobial stewardship practices by determining the prevalence and associated factors for ESBL-producing *E. coli* and *Klebsiella pneumoniae* in hospitalized older adults with upper UTIs.

## Methods

### Study design and participants

This study was conducted in a tertiary-care university hospital, which has 676 active adult beds. A prospective observational study of consecutive hospitalized patients with the diagnosis of community-onset or hospital-acquired UTI was conducted in a tertiary university hospital over a 1-year period from October 2019 to September 2020. All adult patients who were consulted by the Infectious Diseases and Clinical Microbiology Department with the diagnosis of complicated UTI in the emergency department and hospital wards were recorded. Patients >65 years were included in the study if their urinary specimen culture showed monomicrobial isolation of *E.coli* or *Klebsiella pneumoniae* with a colony-forming unit count of ≥10,000/ml. Diagnosis of acute pyelonephritis or sepsis, severe sepsis, or septic shock of urinary origin was based on symptoms and abnormal urinalysis in patients with no other apparent source of concomitant infection.

**Figure 1:**
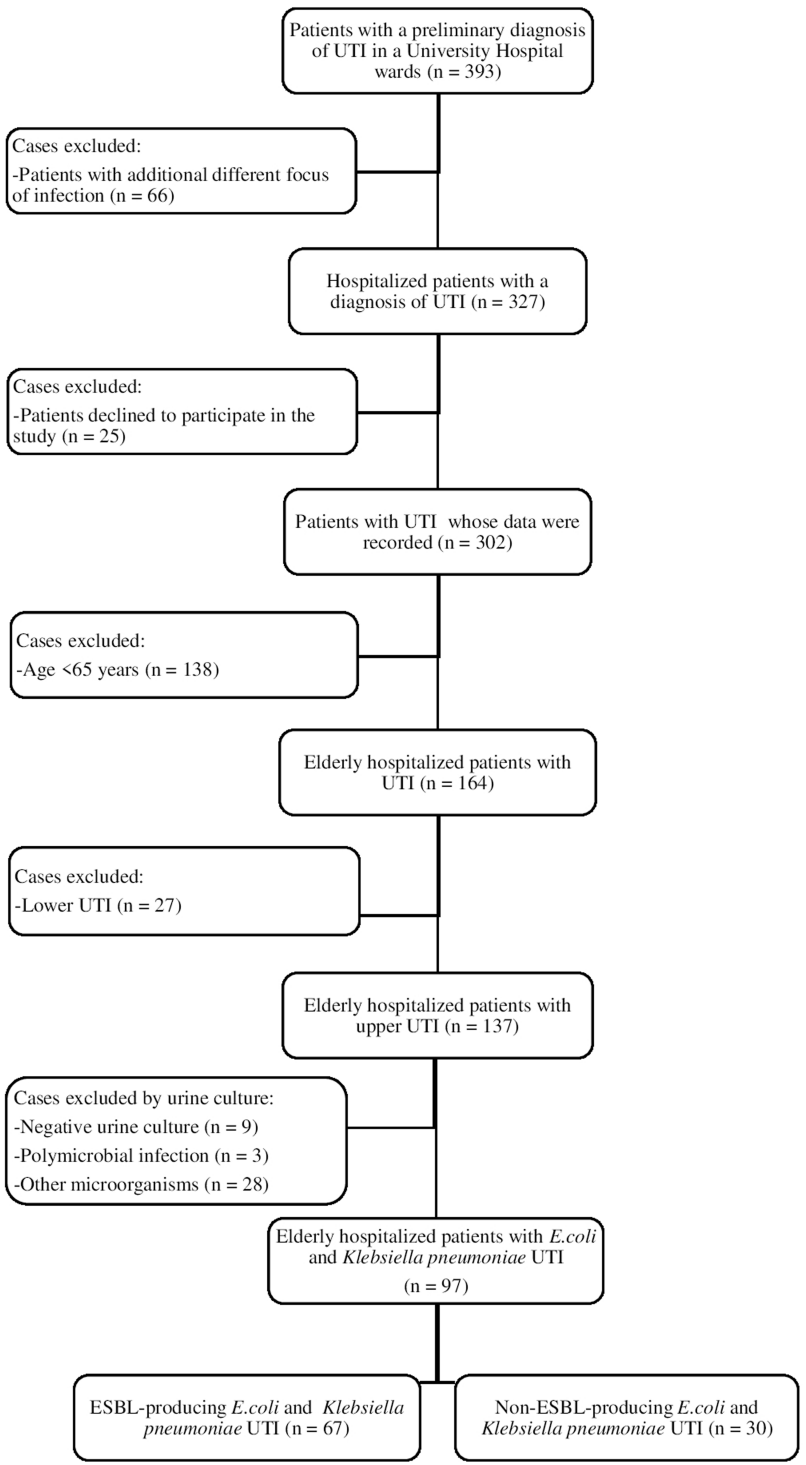
Flowchart of cases included in the study.

In 1 year, 393 adult patients were followed up with the diagnosis of complicated UTIs. A total of 97 of the 393 patients were finally included in the study (Figure 1). All included patients were aged >65 years with *E. coli* or *Klebsiella pneumoniae* upper UTIs.

### Data collection

Demographic data such as age, gender, and comorbidities, predisposing and immunosuppressive conditions, symptoms and physical examination findings, laboratory tests, culture results, antimicrobial treatments, and response to treatment of the patients diagnosed with UTIs were recorded. We verified the past medical histories of the patients through the electronic system of our hospital (ISHOP), the “e-nabiz” electronic system of the Ministry of Health, and the “medeczane” electronic database of the National Reimbursement Agency of our country.

In order to determine the clinical factors predicting ESBL production, cases with *E. coli* and *Klebsiella pneumoniae* isolated in urine cultures were classified according to their ESBL positivity.

Possible associated factors analyzed as independent variables included age, gender, previous recurrent UTIs, comorbidities, clinical findings (fever, vital signs, and acute phase parameters), and clinical severity (bacteremia, acute renal failure, and septic shock). Outcome variables were inappropriate empirical antibiotic treatment (IEAT), recurrence of UTIs, and in-hospital mortality. The cases were followed up for 3 months in terms of relapse/reinfection.

### Definitions

The diagnosis of UTI was established by evaluating the clinical and laboratory findings of the patients according to the IDSA guidelines. Acute pyelonephritis/upper UTI was defined as the involvement of the bladder and kidneys with fever, flank pain, and costovertebral angle tenderness. Urosepsis/severe UTI was defined as an uncontrolled immune response in the host against urinary infection, leading to life-threatening organ failure. Organ failure was evaluated according to the “Sequential Organ Failure Assessment (SOFA)” scoring. In a patient with sepsis, the persistence of hypotension despite vasopressor treatment and serum lactate level > 2 millimoles/liter (mmol/L) despite adequate fluid resuscitation was defined as septic shock.

Secondary bacteremia was defined as the isolation of the same microorganism in both urine and blood cultures with the same antibiotic susceptibility profile.

A UTI was classified as hospital-acquired if the infection onset was either 48 h after hospitalization or within 3 days after discharge.

A history of recurrent UTIs was defined as ≥2 episodes of UTIs within 6 months or ≥ 3 episodes within 12 months, as documented in the patients’ past medical history.

An empirical antimicrobial *in vitro* resistance against the causative microorganism was regarded as IEAT.

### Microbiological methods

Urine and blood culture results were reported in the laboratory of the Infectious Diseases and Clinical Microbiology Department. Urine samples were collected sterile as midstream clean catch or from a urinary catheter. The samples were inoculated onto chromogenic (HiCrome Urinary Tract Infection Agar) media. The blood cultures were performed using the BACTEC (Becton Dickinson, Franklin Lakes, NI, USA) automated system. Isolated organisms were identified according to conventional procedures. Antimicrobial susceptibility of organisms was determined using the Kirby–Bauer disk diffusion method on Mueller–Hinton agar according to European Committee on Antimicrobial Susceptibility Testing (EUCAST) recommendations. Isolates with “intermediate” susceptibility were classified as “resistant” in the final analysis. Confirmation of ESBL positivity (generally characterized by reduced susceptibility to one or more of ceftazidime, cefotaxime, and ceftriaxone) was performed using a double disk synergy test (DDST). The increased inhibitor activity of third-generation cephalosporins in the presence of vicinal clavulanic acid (synergy) indicates the production of ESBL in Gram-negative bacilli.

### Statistical analysis

The chi-square test was used for categorical variables. The Mann–Whitney U-test was used to compare continuous variables. Univariate regression analysis was performed. All variables detected as significant in a univariate analysis were included in the multivariate analysis. Results were evaluated at a 95% confidence interval, and the statistical significance level was defined as a *p*-value of <0.05. The analyses were performed using the IBM Statistical Package for Social Sciences 25 (SPSS-25, Chicago, IL, USA).

## Results

Of 97 patients with UTIs, 54.6% were women, and the mean age was 75.8 ± 7.9 years. Most of the patients had one or more comorbidities. Of the urine samples, 66 were collected via urine clean catch and 31 were obtained via urine catheters. Almost half of the cases were hospital-acquired UTIs. Urosepsis was determined in 73.2% of cases, and septic shock developed in 8.2%. The main demographic and clinical characteristics of the patients are shown in Table 1.

**Table 1:** Characteristics of patients with urinary tract infections caused by extended-spectrum β-lactamase (ESBL)-producing *Escherichia coli* and *Klebsiella pneumoniae* and non-ESBL-producing isolates.

Patient characteristics	All	ESBL	Non-ESBL	*p*-value
	*N* = 97	*n* = 67 (69.1%)	*n* = 30 (30.9%)	
Age years, mean ± SD	75.9 ± 8.0	75.4 ± 7.8	76.8 ± 8.4	0.47
Male gender, no. (%)	44 (45.4)	35 (52.2)	9 (30)	**0.040**
Hospital-acquired UTI, no. (%)	47 (48.5)	31 (46.3)	16 (53.3)	0.520
Hospitalization within the past 3 months, no. (%)	55 (56.7)	43 (64.2)	12 (40)	**0.030**
Antibiotic use within the past 3 months, no. (%)	63 (64.9)	47 (70.1)	16 (53.3)	0.110
Comorbidities, no. (%)				
Diabetes mellitus	42 (43.3)	28 (41.8)	14 (46.7)	0.650
Hypertension	79 (81.4)	54 (81)	25 (83.3)	0.750
Chronic renal disease	36 (37.1)	25 (37.3)	11 (36.7)	0.950
Chronic respiratory disease	19 (19.6)	15 (22.4)	4 (13.3)	0.410
Congestive heart failure	46 (47.4)	30 (44.8)	16 (53.3)	0.440
Immunosuppression	21 (21.6)	15 (22.4)	6 (20)	0.792
Malignancy	20 (20.6)	16 (23.9)	4 (13.3)	0.240
Benign prostatic hyperplasia	27 (27.8)	22 (32.8)	5 (16.7)	0.140
Urolithiasis	15 (15.5)	12 (17.9)	3 (10)	0.320
Urinary tract obstruction	10 (10.3)	9 (13.4)	1 (3.3)	0.131
Prior recurrent UTI	52 (53.6)	41 (61.2)	11 (36.7)	**0.030**
Invasive urological procedure	27 (27.8)	22 (32.8)	5 (16.7)	0.10
Catheter-associated UTI, no. (%)	31 (32)	19 (28.4)	12 (40)	0.260
Fever >38° C no. (%)	45 (46.4)	33 (49.3)	12 (40)	0.400
Heart rate, bpm, mean ± SD	92.8 ± 20.4	93.6 ± 20.4	91.1 ± 20.8	0.440
Systolic blood pressure, mm Hg, mean ± SD	124 ± 30	125 ± 31	122 ± 27	0.742
WBC > 12.000 /mm^3^, no. (%)	40 (41.2)	32 (47.8)	8 (26.7)	0.051
CRP, mg/L, mean ± SD	124 ± 109	147 ± 119	74 ± 60	**0.004**
Bacteremia, no. (%)	20 (20.6)	18 (26.9)	2 (6.7)	**0.020**
Acute renal failure, no. (%)	32 (33)	23 (34.3)	9 (30)	0.680
Septic shock, no. (%)	8 (8.2)	7 (10.4)	1 (3.3)	0.240
Inappropriate empiric antibiotic treatment, no. (%)	23 (23.7)	23 (34.3)	0	**0.001**
Duration of hospitalization before infection (for hospital-acquired UTIs), day, mean ± SD	15.5 ± 15.1	16.9 ± 15.6	12.7 ± 14.3	0.280
The reasons for the patient’s hospitalization (for hospital-acquired UTIs), no. (%)				0.551
Acute kidney injury	4 (4.1)	2 (3)	2 (6.7)	
Benign prostate hyperplasia	4 (4.1)	2 (3)	2 (6.7)	
Urolithiasis	4 (4.1)	2 (3)	0 (0)	
General surgical procedures	4 (4.1)	2 (3)	2 (6.7)	
Hematological malignancy	5 (5.2)	4 (6)	1 (3.3)	
Chronic artery diseases	5 (5.2)	3 (4.5)	2 (6.7)	
Chronic kidney disease	6 (6.2)	3 (4.5)	3 (10)	
Diabetes mellitus	1 (1)	1 (1.5)	0 (0)	
Polyneuropathie	1 (1)	1 (1.5)	0 (0)	
Rheumatological diseases	2 (2.1)	0 (0)	2 (6.7)	
Cerebrovascular disease	7 (7.2)	5 (7.5)	2 (6.7)	
Malignancy	2 (2.1)	2 (3)	0 (0)	
The antibiotics that the patient received during hospitalization, no. (%)				0.142
Amikacin	1 (1)	1 (1.5)	0 (0)	
Ciprofloxacin	3 (3.1)	2 (3)	1 (3.3)	
Ertapenem	15 (15.5)	12 (17.9)	3 (10)	
Fosfomicin	14 (14.4)	5 (7.5)	9 (30)	
Imipenem/Meropenem	22 (22.7)	18 (26.9)	4 (13.3)	
Levofloxacin	1 (1)	1 (1.5)	0 (0)	
Piperacillin-tazobactam	20 (20.6)	15 (22.4)	5 (16.7)	
Sefoperazon-sulbactam	1 (1)	1 (1.5)	0 (0)	
Ceftriaxone	20 (30.6)	12 (17.9)	8 (26.7)	
Inappropriate empirical antibiotic treatment (IEAT), no. (%)				–
Ciprofloxacin	1 (1)	1 (1.5)	0 (0)	
Ertapenem	1 (1)	1 (1.5)	0 (0)	
Fosfomicin	1 (1)	1 (1.5)	0 (0)	
Imipenem/Meropenem	2 (2.1)	2 (3)	0 (0)	
Levofloxacin	1 (1)	1 (1.5)	0 (0)	
Piperacillin-tazobactam	4 (4.2)	4 (6)	0 (0)	
Sefoperazon-sulbactam	1 (1)	1 (1.5)	0 (0)	
Ceftriaxone	12 (12.4)	12 (17.9)	0 (0)	

Bold values represent the statistically significant results.

The antibiotics given for the prior infections were ciprofloxacin (*n* = 20, 20.6%), cefixime (*n* = 12, 12.4%), cefuroxime (*n* = 11, 11.3%), ceftriaxone (*n* = 14, 14.4%), and amoxicillin/clavulanate (*n* = 6, 6.2%).

*E. coli* was present in 64 patients, and *Klebsiella pneumoniae* was present in 33 patients. ESBL rates (64% vs. 78.8%, *p* = 0.137) and demographic characteristics of patients with *E. coli* and *Klebsiella pneumoniae* were similar.

ESBL prevalence among UTIs with *E. coli* or *Klebsiella pneumoniae* was 69.1% (*n* = 67). Male gender (52.2% vs. 30%), hospitalization within the past 3 months (64.2% vs. 40%), prior recurrent UTIs (61.2% vs. 36.7%), and secondary bacteremia (26.9% vs. 6.7%) in the follow-up were more prevalent in the ESBL-producing group than in the non-ESBL-producing group. Leukocytosis (leukocyte count >12.000/mm^3^) was detected more frequently in the ESBL-producing group (47.8% vs. 26.7%, *p* = 0.051). CRP values at the time of UTI diagnosis were found to be significantly higher in the ESBL-producing group. The mean CRP was 146.8 mg/L in the ESBL-producing group and 73.5 mg/L in the non-ESBL-producing group (*p* = 0.004). The IEAT rate was observed as 34.3% in the ESBL-producing group and none in the non-ESBL-producing group. The rate of in-hospital mortality was 3.1%. All three deaths were in the ESBL-producing group. The difference between the rates of post-treatment recurrence in ESBL producers (38.8%) and non-ESBL producers (46.7%) was not statistically significant (*p* = 0.470).

The empirical antibiotics used for treatment were: meropenem in 22 (22.7%) cases, with 4.5% being IEAT; ceftriaxone in 20 (20.6%), with 60% being IEAT; piperacillin-tazobactam in 20 (20.6%), with 25% being IEAT; ertapenem in 14 (14.4%), with 7.1% being IEAT; fosfomycin in 15 (15.5%), with 6.7% being IEAT; ciprofloxacin/levofloxacin in 4 (4.1%), with 50% being IEAT; amikacin in 1 (1%), which was appropriate; and cefoperazone sulbactam in 1 (1%), which was IEAT.

Antimicrobial resistance profiles of *E. coli* and *Klebsiella pneumoniae* isolates in accordance with major epidemiological variables are provided in Table 2. Ertapenem and meropenem were the two antibiotics with resistance rates of less than 20% in the whole cohort. Piperacillin-tazobactam and amikacin resistance rates were 21.9 and 22.6%, respectively.

**Table 2:** Antimicrobial resistance profiles of urinary *E. coli* and *Klebsiella* spp. isolates in accordance with major epidemiological groups.

Criteria	AMP	AMC	CIP	SXT	AK	CRO	CTX	CAZ	TZP	ETP	MEM
All isolates *N*: 97	91/96 94.8%	63/95 66.3%	57/97 58.8%	56/93 60.2%	21/93 22.6%	62/94 66%	56/89 62.9%	61/97 62.9%	21/96 21.9%	12/96 12.5%	8/97 8.2%
ESBL (*p*-value)	**0.001**	**0.001**	**0.001**	**0.003**	**0.002**	**0.001**	**0.001**	**0.001**	**0.001**	**0.013**	**0.048**
Yes (*n* = 67)	67/67 100%	53/65 81.5%	49/67 73.1%	46/66 69.7%	20/63 31.7%	61/64 95.3%	55/59 93.2%	59/67 88%	21/66 31.8%	12/66 18.2%	8/67 11.9%
No (*n* = 30)	24/29 82.8%	10/30 33.3%	8/30 26.7%	10/27 37%	1/30 3.3%	1/30 3.3%	1/30 3.3%	2/30 6.6%	0/30 0%	0/30 0%	0/30 0%
Hospital-acquired UTI (*p*-value)	0.14	0.83	0.875	0.137	0.677	0.559	0.217	0.259	0.179	**0.045**	**0.021**
Yes (*n* = 47)	42/46 91.3%	31/46 67.4%	28/47 60%	30/44 68.2%	11/45 24.4%	29/46 63%	26/47 55.3%	30/47 63.8%	13/47 27.7	9/46 19.6%	7/47 14.9%
No (*n* = 50)	49/50 98%	32/49 65.3%	29/50 58%	26/49 53.1%	10/48 20.8%	33/48 68.7%	30/50 60%	31/50 62%	8/49 16.3%	3/50 6%	1/50 2%
Prior recurrent UTI (*p*-value)	0.514	0.217	0.312	0.583	0.178	**0.028**	0.219	0.070	0.421	0.757	0.831
Yes (*n* = 52)	50/52 96.2%	36/50 72%	33/52 63.5%	32/51 62.7%	14/50 28%	38/50 76%	33/52 63.5%	37/52 71.2%	13/52 25%	7/52 13.5%	4/52 7.7%
No (*n* = 45)	41/44 93.2%	27/45 60%	24/45 53.3%	24/42 57.1%	7/43 16.3%	24/44 54.5%	23/45 51.1%	24/45 53.3%	8/44 18.2%	5/44 11.4%	4/45 8.9%

AMP, ampicillin; AMC, amoxicillin/clavulanic acid; CIP, ciprofloxacin; SXT, trimethoprim/sulfamethoxazole; AK, amikacin; CRO, ceftriaxone; CTX, cefotaxime; CAZ, ceftazidime; TZP, piperacillin/tazobactam; ETP, ertapenem; MEM, meropenem. Bold values represent the statistically significant results.

The multivariate analysis revealed that male gender (OR: 2.72, CI: 1.02–7.25), prior recurrent UTI (OR: 3.14, CI: 1.21–8.14), and development of secondary bacteremia (OR 4.95, CI 1.03–23.89) were major associated factors for UTIs in the older adults due to ESBL-producing *E. coli* and *Klebsiella pneumoniae*.

## Discussion

In this study, we found that male gender, recurrent UTIs, and secondary bacteremia were independent associated factors for acquiring an ESBL resistance profile in hospitalized older adults with *E. coli* and *Klebsiella pneumoniae* UTIs. Prior hospitalization was significant in the univariate analysis, but the multivariate analysis did not reveal it as an associated factor for ESBL UTIs.

In a recent study on hospitalized older adults, Artero et al. identified recurrent UTIs and healthcare-associated UTIs (hospitalization within 3 months, antibiotic use within 3 months, or nursing home residence) as associated factors for ESBL-positive UTIs (Artero et al., 2017).

Many previous studies have similarly documented being male as a predictor of ESBL urinary infection in adults (Nakai et al., 2016; Lee et al., 2018). A recent retrospective study from China, which included a case series where 56% of the participants were over 60 years of age, showed that male gender, older age, hospital stay within the preceding 3 months, invasive urological procedure, and antibiotic use within the previous 3 months were independent factors associated with the development of an ESBL-positive Enterobacteriaceae UTI (Liu et al., 2022). Similar risk factors, such as diabetes mellitus, recurrent UTIs, age over 60, and male gender, were also described by Rodríguez-Bano and Pascual (2004).

Despite many studies identifying a history of antibiotic exposure (IV or oral) as an associated factor for ESBL-positive UTI, we were not able to document recent exposure to antibiotics as an associated factor for infection with ESBL in our study, similar to a recently published study (Hertz et al., 2016). One reason for this may be the high rates of antibiotic use in both ESBL-positive and non-ESBL groups.

The prevalence of ESBL organisms varies by geographical region, ranging from 5% in Japan to 31% in China and 36% in some European countries, as well as approximately 40% in Egypt and Pakistan (Hanberger et al., 1999; Al-Zarouni et al., 2008; Nakai et al., 2016; Abrar et al., 2018; Ranjan Dash et al., 2018; Shash et al., 2019). In the United States, a multicenter prospective study found the prevalence of ESBL-producing Enterobacterales among UTI patients to be 17.2% (Lee et al., 2018).

According to the data of our country’s National Antimicrobial Resistance Surveillance System, the rates of ESBL *E. coli* were 44.54, 44.61, and 54.53%, and ESBL *Klebsiella pneumoniae* rates were 52.13, 50.24, and 66% in healthcare-associated infections in Turkey in 2019, 2020, and 2021, respectively (National Antimicrobial Resistance Surveillance System, 2021). Accordingly, in our study, we found an alarmingly high percentage of ESBL-producing *E. coli* and *Klebsiella pneumoniae* in hospitalized older adults with UTIs (69.1%). Tüzün T. et al. from Turkey also reported that 50.5% of the cases were infected with ESBL-producing *E. coli* in their prospective cohort study of community-onset UTIs caused by *E. coli* between 2012 and 2014 (Tüzün et al., 2019).

Similar to a very recently published study, we found a high rate of ESBL positivity and resistance to other antibiotic classes (fluoroquinolones, sulfonamides, and aminoglycosides) in both community-onset and hospital-acquired UTI cases (Herbawi et al., 2024). Carbapenem resistance was also detected in 11.9% of cases with ESBL positivity. We assume that this is due to the clinical complexity of our cases as well as the fact that we are a high ESBL prevalence country, with similar rates reported from India (Tankhiwale et al., 2004).

ESBL production has been identified as a significant risk factor associated with mortality in systemic infections due to Enterobacterales (Kang et al., 2005; Tumbarello et al., 2006). In their study, Nam Su Ku et al. found that previous antimicrobial therapy and a high initial SOFA score were independent risk factors for 30-day mortality in older adults infected with ESBL-positive agents, but the urinary source of infection was an independent determinant for non-mortality (Ku et al., 2014). In our study, although the mortality rate in the ESBL group (4.5%) was higher compared to the non-ESBL group, we did not find it statistically significant.

We found that the rates of post-treatment recurrent UTIs were also similar in both ESBL and non-ESBL groups. One reason may be that the patients were followed prospectively and carbapenem treatment was initiated promptly in cases of sepsis. In a multicenter study conducted in Sweden, recurrent infection, diabetes mellitus, and urogenital disease were associated with relapse in UTIs caused by ESBL-producing Enterobacterales (Montelin et al., 2024).

In our cases, we found secondary bacteremia significantly higher in the ESBL producers. Switching directly to carbapenem therapy should be considered when secondary bacteremia is detected.

This study has several limitations. First, the sample size was small to include more variables in the multivariate analysis. Second, only inpatients were recruited. Therefore, our study group may not be representative of the greater population.

In conclusion, severe UTIs in older men with a history of recurrent UTIs may serve as a warning to clinicians regarding the potential for ESBL production, especially in settings with a high prevalence of ESBL. These risk factors should be taken into account in the management of empirical treatment. Carbapenems may be prioritized in the empirical treatment of patients with known risk factors for ESBL.

## Data availability statement

The raw data supporting the conclusions of this article will be made available by the authors, without undue reservation.

## Ethics statement

The studies involving humans were approved by Istanbul University-Cerrahpasa, Cerrahpasa Medical Faculty, Clinical Research Ethics Committee. The studies were conducted in accordance with the local legislation and institutional requirements. The participants provided their written informed consent to participate in this study.

## Author contributions

SA: Conceptualization, Formal analysis, Methodology, Project administration, Validation, Writing – original draft, Writing – review & editing, Data curation, Funding acquisition, Investigation, Resources, Software, Visualization. IB: Supervision, Validation, Writing – original draft, Writing – review & editing. SS: Formal analysis, Validation, Writing – original draft, Writing – review & editing. OB: Formal analysis, Funding acquisition, Investigation, Methodology, Project administration, Resources, Software, Validation, Visualization, Writing – original draft, Writing – review & editing. SK: Writing – original draft, Writing – review & editing. RK: Supervision, Writing – original draft, Writing – review & editing. BM: Supervision, Writing – original draft, Writing – review & editing. GA: Supervision, Writing – original draft, Writing – review & editing. FT: Supervision, Writing – original draft, Writing – review & editing. NS: Conceptualization, Formal analysis, Methodology, Project administration, Supervision, Validation, Writing – original draft, Writing – review & editing.
